# Experimental investigation of mechanical properties of sustainable silica sand reinforced AA6061 composites subjected to thermomechanical treatment

**DOI:** 10.1038/s41598-025-20553-1

**Published:** 2025-10-21

**Authors:** Ramakrishna Vikas Sadanand, Suhas Yeshwant Nayak, Aruna Prabhu

**Affiliations:** Department of Mechanical and Industrial Engineering, Manipal Institute of Technology, Manipal Academy of Higher Education, Manipal, 576104 Karnataka India

**Keywords:** AA6061 composites, Silica sand, Stir casting, Thermomechanical treatment, Mechanical properties, Sustainability, Structural materials, Characterization and analytical techniques, Design, synthesis and processing, Imaging techniques, Microscopy

## Abstract

This study investigates the mechanical performance of sustainable AA6061 matrix composites reinforced with silica sand and subjected to thermomechanical treatments. The objective of this study is to assess silica sand as a cost-effective, eco-friendly alternative to traditional ceramic reinforcements in enhancing strength, hardness, and durability. Given the growing demand for sustainable materials in aerospace, automotive, and structural applications, this research explores the potential of silica sand to improve composite properties. The hypothesis is that silica sand, when uniformly dispersed in the AA6061 matrix and processed through rolling and peak ageing, can significantly enhance the mechanical properties. Composites were fabricated via stir casting with 2%, 4%, and 6% silica sand by weight, followed by low-temperature thermomechanical treatment. Mechanical testing included Brinell and Vickers hardness tests, tensile strength evaluation, microstructural analysis, and fracture surface examination. The results revealed consistent improvements with increased reinforcement. Compared to the as-cast composite, the 6% silica sand composite treated at 100 °C with 15% deformation exhibited a 118% increase in hardness and a 62% inrease in tensile strength. Fracture analysis revealed a mixed mode with predominantly brittle failure after treatment. These findings confirm that the combination of silica sand with suitable processing, can produce high-performance, sustainable aluminium composites.

## Introduction

The increasing demand for advanced materials in aerospace, automotive, and structural applications has spurred significant interest in the development of metal matrix composites (MMCs) due to their exceptional mechanical properties, thermal stability, and resistance to wear and corrosion^1,2^. Aluminium alloy 6061 (AA6061), renowned for its excellent machinability, corrosion resistance, and high strength-to-weight ratio, has emerged as a widely used matrix material for MMCs^3–5^. The introduction of ceramic reinforcements into AA6061 has enabled the fabrication of composites with enhanced mechanical performance, tailored for specific engineering applications. Over the years, a variety of ceramic reinforcements, including silicon carbide (SiC)^6–8^, alumina (Al₂O₃)^9,10^, boron carbide (B₄C)^8,11–13^, titanium carbide (TiC)^14–17^ and titanium dioxide (TiO₂), have been extensively explored. These reinforcements significantly improve the hardness, tensile strength, and wear resistance of AA6061 composites by altering the microstructure and introducing strong interfacial bonding between the matrix and the reinforcement. While these materials have demonstrated remarkable results, silica sand as a reinforcement presents a novel and economically attractive alternative. Silica sand, which is abundant and cost-effective, offers comparable mechanical and thermal properties that make it a viable candidate for sustainable composite development.

Zuhailawati et al. produced and tested aluminium composites with silica particulates prepared by powder methodology. Two types of silica, i.e., commercially available silica and silica sand, were used as reinforcements and 99.8% aluminium was used as the matrix material. The reinforcement particles were added at compositions of 0, 10, 20, 30 and 40 vol% and blended in a planetary mill for 2 h, pressed at 200 MPa, and then sintered at a fixed temperature of 600 °C for 5 h under flowing argon gas. The tests revealed that increasing the silica particulates by up to 30% led to an improvement in hardness and fracture toughness. Furthermore, the addition of silica sand over commercial silica resulted in better properties^18^. Rohatgi et al. examined the tribological performance of silica sand particle-reinforced AA206 alloy MMCs. The stir casting technique was followed to add 9 and 13 wt% silica sand particles to produce the composites. To improve the wettability, 5 wt% magnesium was added to the melt along with silica sand particles under the argon. The heat treatment process consisted of solutionizing at 510–516 °C for 2 h, 526–530 °C for 14–20 h, water quenched and artificially aged at 199 °C for 8 h. The wear test revealed a higher wear rate with increasing silica sand content and applied pressure. The composite heat treated to T6 condition did not significantly decrease the wear rate, and the temperature in the vicinity of the counter surface increased with increasing in reinforcement particles^19^. Hemanth fabricated and tested AA356 MMCs reinforced with fused silica particles. The composites were cast with 3, 6, 9, and 12% fused silica reinforcements of 50–100 μm average particle size dispersed in the matrix. The composites were cast by incorporating the fused silica (preheated at 500 °C) into the molten metal and then poured into sand-cast moulds. The microstructure study revealed uniform dispersal of the reinforcement with no agglomeration. The characterization revealed an increase in hardness, tensile, and wear properties with increasing reinforcement content, with the best properties obtained at 9% addition of reinforcement^20^. Moghadam et al. investigated the effect of incorporating foundry silica sand into the aluminium alloy to produce composites with magnesium as a wetting agent. The composites were fabricated by incorporating silica sand particles (100 to 250 μm) and Mg turnings preheated to 120 °C into AA206 using the stir casting route. The study of microstructure and SEM analysis revealed proper wetting of the reinforcing particulates with the addition of Mg and the formation of various reaction products like Al_2_O_3_ and MgAl_2_O_4_. The hardness study indicated increased properties with the increased concentration of silica sand, which had better properties in the case of adding Mg, resulting in better bonding between the particulates and the matrix^21^.

In this study, the focus is on the experimental investigation of silica sand-reinforced AA6061 composites subjected to thermomechanical treatment. By drawing upon the principles established through research on other ceramic-reinforced composites, this work seeks to explore how silica sand despite its relatively untapped potential can serve as a reinforcement to further enhance the mechanical properties of AA6061. Silica sand is a valuable reinforcement material for hybrid metal matrix composites because of its cost-effectiveness and wide availability. It offers excellent thermal stability, enhancing the performance of composites at high temperatures. Silica sand improves wear resistance, making composites more durable for demanding applications. Its lightweight nature helps reduce the overall weight of the composite material without compromising its strength. Additionally, silica sand is easy to process and incorporate into the metal matrix, simplifying manufacturing. These properties make silica sand an attractive and practical choice for reinforcing metal matrix composites, balancing performance, cost, and ease of use. This approach not only promotes the utilisation of locally available materials but also addresses the growing need for sustainable and environmentally conscious solutions in materials science. To achieve this goal, the study involves subjecting the composites to thermomechanical treatments such as rolling and peak ageing. These processes are critical for optimising the microstructure, refining grain boundaries, and enhancing interfacial bonding, thereby improving the mechanical properties of the composite. Key properties such as hardness and tensile strength will be evaluated to assess the effectiveness of silica sand reinforcement and the role of thermomechanical treatments.

Moreover, fracture surface analysis of tensile specimens will be carried out to provide insights into the microstructural transformations induced by peak ageing and rolling. By examining the fracture surfaces, this analysis aims to elucidate the mechanisms underlying crack propagation, fracture modes, and the influence of treatment processes on the mechanical behaviour of composites. These findings are expected to offer a deeper understanding of the interplay between reinforcement, thermomechanical treatment, and mechanical properties, paving the way for the development of high-performance composites. In conclusion, this research endeavours to bridge the knowledge gap in silica sand-based composites by systematically investigating their mechanical performance and microstructural characteristics. The outcomes of this study hold significant potential for advancing the field of composite materials, contributing to the development of cost-effective, sustainable, and high-performance solutions for industrial applications.

## Methodology

### Materials

Wrought AA6061, containing 0.77% Si, 0.92% Mg, 0.22% Fe, 0.27% Cu, 0.06% Mn, 0.07% Cr, and 0.02% Ti by weight, was selected as the matrix alloy for this study. The composition of the alloy was determined through spectroscopy analysis. AA6061 is well-regarded for its excellent strength-to-weight ratio, corrosion resistance, and good machinability, making it an ideal candidate for use in a variety of composite materials^22–25^. In this research, silica sand was employed as the reinforcing material to fabricate AA6061-based MMCs. The silica sand was obtained from the Swarna river basin deposits near Manipal. The extracted sand containing impurities such as organic matter, silt and clay was washed with water multiple times. The surface of the sand was cleaned with distilled water and acetone with the help of an ultrasonic agitator. The agitation process involved three 15 min cycles with distilled water, followed by a single cycle with acetone. The acetone was removed by decantation, and the particles were dried in a hot air oven at 200 °C to eliminate moisture After drying, the silica sand particles were sized using a ball mill. Furthermore, the particles were sieved to sort the particles to 50–75 μm average particle size. The particle size range was carefully selected to ensure uniform distribution within the matrix and optimal interfacial bonding.

### Fabrication of the MMCs

As part of the study, AA6061 aluminium alloy billets were sourced and cut into smaller sizes before being heated to a molten state in an electrical resistance-type furnace at 750 °C. The molten metal was carefully processed to remove impurities, which were skimmed off as slag to ensure the purity of the matrix alloy. Preheated silica sand particulates, sieved to sizes between 50 and 75 μm and maintained at 500 °C, were gradually added into the vortex formed in the molten alloy by mechanical stirring. The stirring process was carried out using a three-blade mild steel stirrer operating at speeds of 200–300 rpm, ensuring a consistent and well-formed vortex to facilitate the uniform incorporation of the silica sand particulates into the molten alloy.

**Fig. 1:**
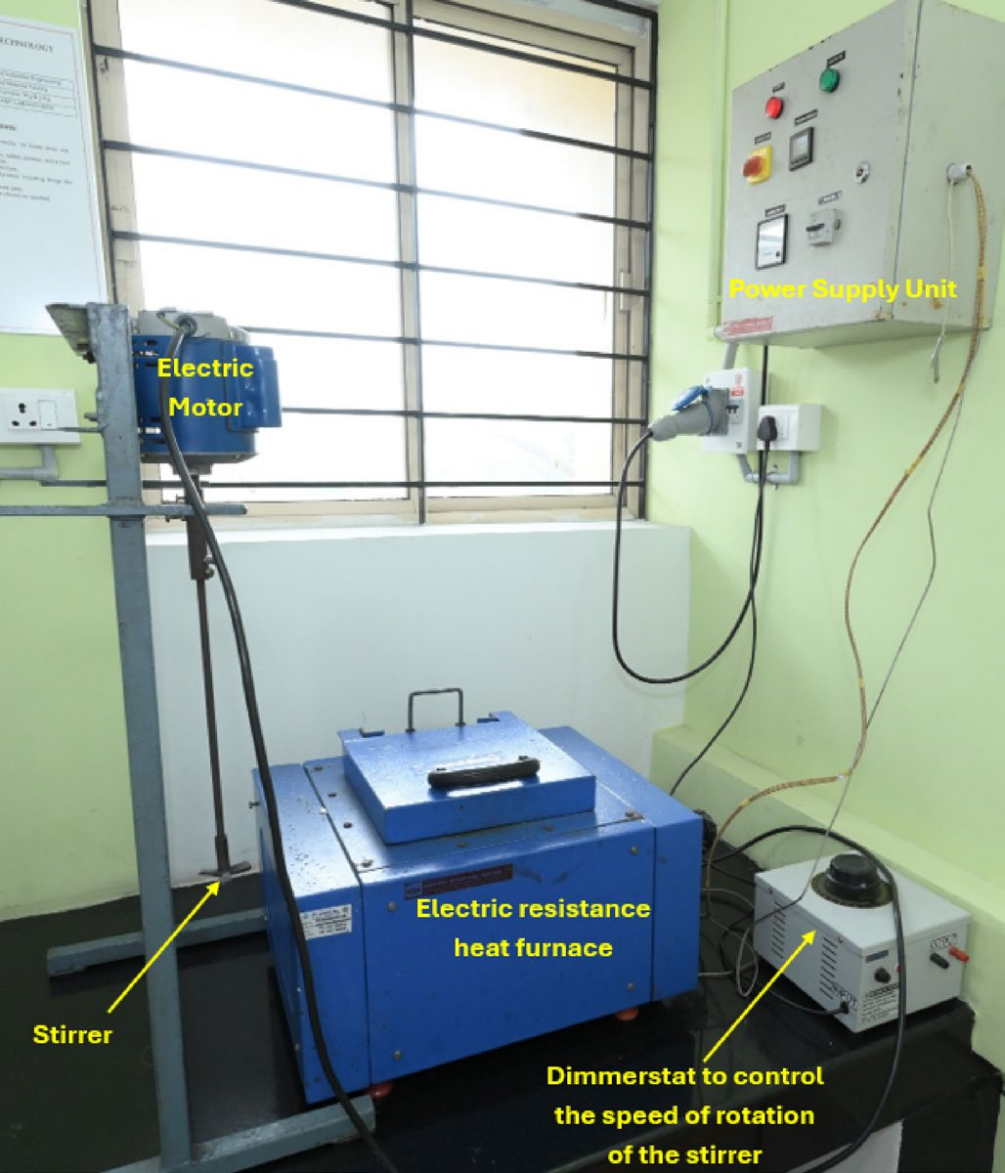
Stir casting equipment used for the fabrication of AA6061-silica sand composites.

The incorporation of silica sand particulates was performed with three different weight fractions, namely 2%, 4%, and 6%, to fabricate the AA6061-silica sand composites. After the reinforcement particles were added through the vortex, the composite mixture was stirred mechanically for an additional 5 min to ensure homogeneity and effective dispersion of the silica sand within the aluminium matrix. The molten composite was then maintained at an elevated temperature of 760 °C and subsequently poured into preheated permanent moulds, which were maintained at 500 °C to avoid thermal shock and ensure controlled solidification. The composite materials were allowed to cool and solidify, completing the stir-casting fabrication process. Figure 1 shows the stir casting setup used for the fabrication of the composites. The incorporation of sieved silica sand into the AA6061 matrix through the stir casting technique provides an innovative avenue for exploring the potential of aluminium-based composites for various industrial applications. This unique combination of silica sand reinforcement and the intrinsic properties of the matrix alloy is expected to produce composites with superior mechanical performance, making them suitable for high-stress environments such as aerospace structures, automotive components, and other demanding engineering applications.

### Machining and specimen preparation

The as-cast composite specimens were first machined and cut into dimensions of 120 mm × 40 mm × 15 mm. Subsequently, these specimens were further processed and machined into strips with dimensions of 120 mm × 4 mm × 15 mm. These strips were then precisely machined to produce specimens of three different thicknesses, as outlined under the “Initial Thickness” column in Table 1. All the cutting and machining operations were carried out using an Abrasive Water Jet (AWJ) machine, ensuring high precision and accuracy. Cubical specimens measuring 10 mm × 10 mm × 15 mm were extracted from the as-cast composites for microstructure analysis. To achieve a smooth and reflective surface, each specimen was meticulously polished along the flat surface using silicon carbide sandpapers of varying grit sizes. The polishing process was carried out sequentially with grit sizes of 80, 100, 220, 400, 600, 1000, 1500, 2000, and 2500 to progressively refine the surface finish. Following this, the specimens underwent further polishing using a selvyt cloth and diamond suspensions of particle sizes 3 μm, 1 μm, and 0.25 μm, applied sequentially. This final stage of polishing was performed on a rotary disc-type polishing machine to ensure an optimal surface finish suitable for microstructure examination was obtained.

### Treatment of composite specimens

**Fig. 2:**
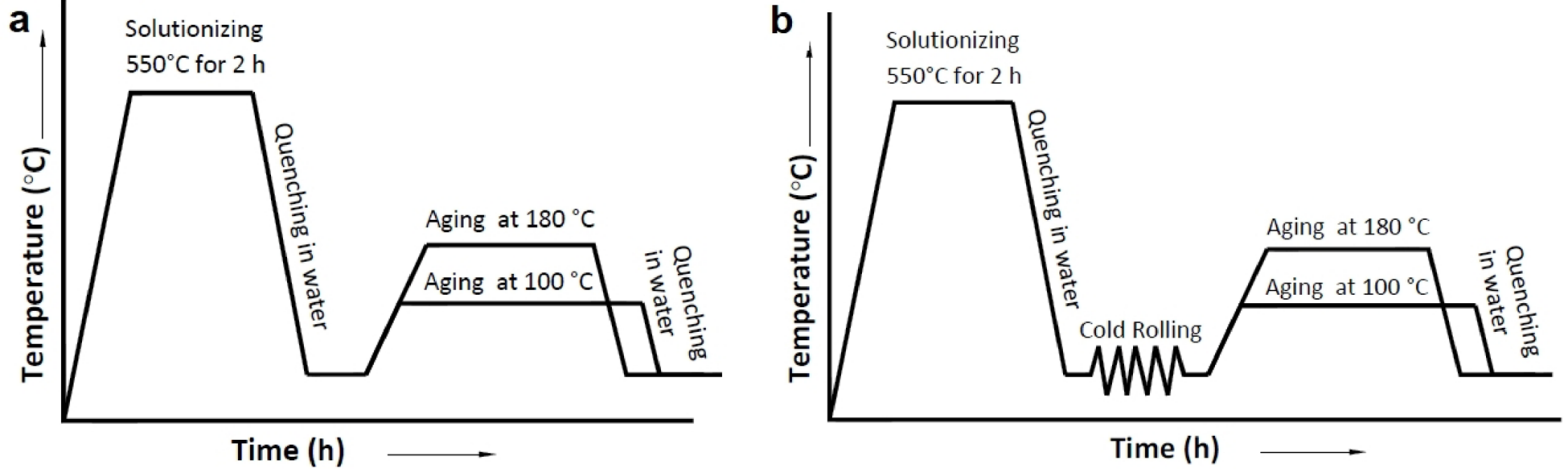
The heat treatment cycles employed to heat treat the AA6061-silica sand composites (**a**) age-hardening and (**b**) low-temperature thermomechanical treatment.

The ageing treatment process (Fig. 2a) involved heating the specimens to a temperature of 550 °C and maintaining them at this temperature for 2 h. Following heating, the specimens were rapidly quenched in ambient temperature water to lock the microstructure in its solution-treated state. The specimens subsequently underwent precipitation hardening at two different temperatures, 100 °C and 180 °C, for varying durations ranging from 1 to 20 h, allowing the formation of precipitates to enhance their mechanical properties.

The low-temperature thermomechanical treatment (LTMT) process (Fig. 2b) similarly began with heating the specimens at 550 °C for 2 h, followed by immediate quenching in ambient temperature water. Post-quenching, the specimens were subjected to cold rolling to induce deformation levels of 5%, 10%, and 15%. This step was implemented to refine the microstructure and promote strain hardening. After cold rolling, the specimens were precipitation hardened at 100 °C and 180 °C for various durations between 1 and 20 h to evaluate the influence of thermomechanical processing on the mechanical behaviour of the material. This systematic approach is aims to understand the interplay between thermal and mechanical processing techniques and their effects on the microstructure and properties of the materials.

### Extraction of reinforcement particles from silica sand-reinforced MMCs

**Fig. 3:**
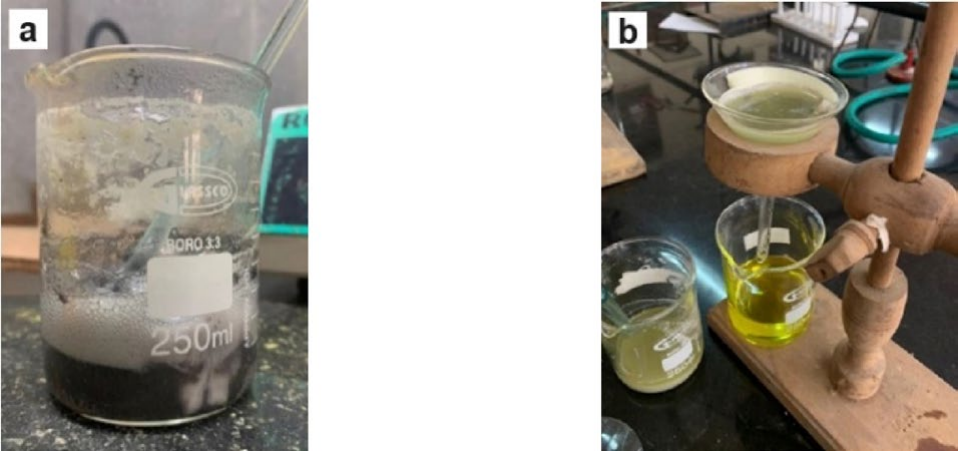
Extraction of silica sand particles from the composite which involves (**a**) dissolution of AA6061 in HCl and (**b**) filtration of particles using Whatman filter paper.

Chemical analysis was conducted to verify the presence and weight percentages of the added reinforcement particles in the composites. The process involved dissolving the aluminium alloy from the composite specimen in concentrated hydrochloric acid (HCl). To prepare for the analysis, the composite specimens were first preheated to 100 °C to eliminate moisture. Each specimen was weighed and placed into a glass beaker, where concentrated HCl was gradually added. The solution was heated to boiling until the aluminium alloy had completely dissolved. The dissolved alloy left behind the reinforcement particles, which were separated by filtration with Whatman filter paper. The filtered particles were transferred to a hot air oven for drying. Finally, the dried reinforcement particles were weighed, and the difference between their weight and that of the original cast specimen was used to determine the reinforcement weight fraction in the composite. The steps involved in silica sand particle extraction are illustrated in Fig. 3.

### Evaluation of density and void fraction

The theoretical and experimental densities were evaluated to estimate the porosity introduced during the fabrication process. To estimate the theoretical densities (ρ_t_), the Agarwal and Broutman Eq. 2^6^ (Eq. 1) was used while the actual densities (ρ_a_), were determined using a digital density balance (Contech make). The void fraction ($\:{V}_{f})$was then estimated using Eq. 2.

1 $$\:{\rho\:}_{t}=\:\frac{1}{\left(\frac{{w}_{m}}{{\rho\:}_{m}}\right)+\:\left(\frac{{w}_{r}}{{\rho\:}_{r}}\right)}$$

where, $\:{w}_{m}\:and\:{w}_{r}$ - weight fractions of the matrix and reinforcement respectively; $\:{}_{m}\:and\:{}_{r}$ - densities of the matrix and reinforcement respectively

2 $$\:{V}_{f}=\frac{\left({\rho\:}_{t}-{\rho\:}_{a}\right)}{{\rho\:}_{t}}\:\times\:100\%$$

### Evaluation of hardness and tensile strength

**Fig. 4:**
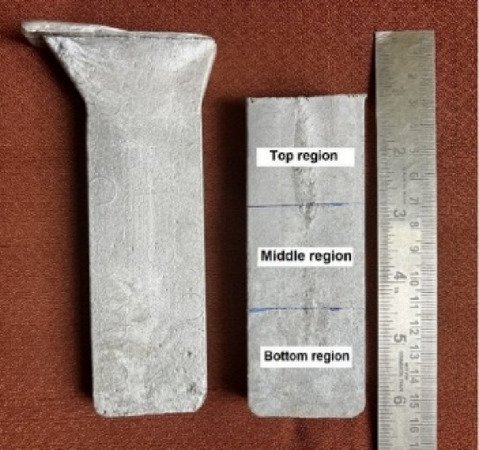
As-cast composite slab with the demarcation showing the 3 regions.

The surface hardness of the as-cast composites was evaluated using a Brinell hardness tester to confirm the uniform distribution and incorporation of reinforcement particles within the matrix. The hardness tests were conducted under ASTM E10–18 conditions^27^. For accuracy, the cast composite specimens, measuring 120 mm × 40 mm × 15 mm, were divided into three distinct regions (illustrated in Fig. 4). An average of five hardness readings was recorded for each specimen to ensure consistency and reliability in the findings.

**Fig. 5:**
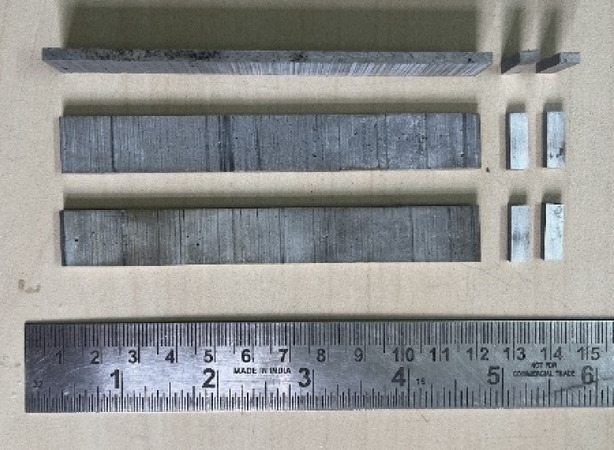
Machined composite specimens for the heat treatment process.

The peak hardness of the heat-treated composites was measured using a Vickers hardness tester, following the ASTM E384 standard^28^. The cast specimens (Fig. 4) were cut to specific dimensions in preparation for heat treatment, followed by peak hardness testing. The specimens were cut into 120 mm × 15 mm × 4 mm strips, as shown in Fig. 5. These strips were then machined to reduce the thickness of each specimen from 4 mm to three different thicknesses, as listed in Table 1 as the initial thickness. The specimens were rolled at room temperature to reduce the thickness by 5, 10, and 15%, respectively. The rolled specimens were further sliced into smaller rectangular-shaped specimens with dimensions of 4 mm × 15 mm × 3 mm. Multiple readings were taken at five distinct locations on the surface of each specimen to guarantee the reliability of the results. These measurements provided insight into the effects of heat treatment and the role of reinforcement on the hardness of the composites.

**Table 1:** Details of the specimen subjected to age-hardening and thermomechanical treatment.

Type of treatment	The initial thickness of the specimen before treatment (mm)	The final thickness of the specimen after treatment (mm)	Average deformation (%)
Age-hardening	3.00	3.00	00
Low-temperature thermomechanical treatment	3.16	3.00	05
	3.33	3.00	10
	3.53	3.00	15

**Fig. 6:**
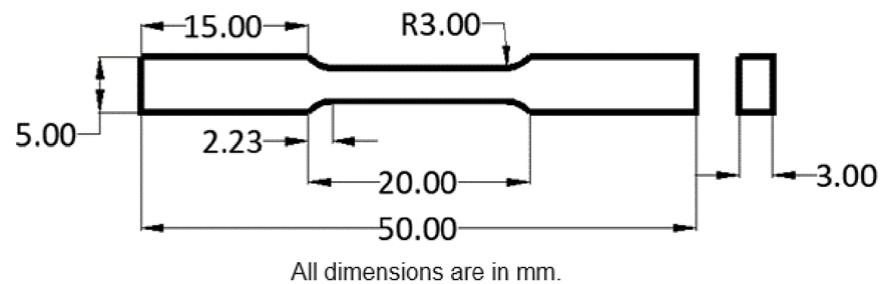
ASTM B557M–15 dimensions for tensile specimens.

The Ultimate Tensile Strength (UTS) of the composites under peak-aged conditions was measured using a horizontal universal testing machine. The tensile test specimens were prepared in accordance with ASTM standard B557M–15^29^, and their dimensions are shown in Fig. 6. To ensure reliable results, an average of three readings was considered for each composite. These tensile strength measurements shed light on the mechanical behaviour of the composite under applied stresses, revealing the effectiveness of the reinforcement and heat treatment processes.

## Results and discussion

### Microstructure and bulk hardness analysis

**Fig. 7:**
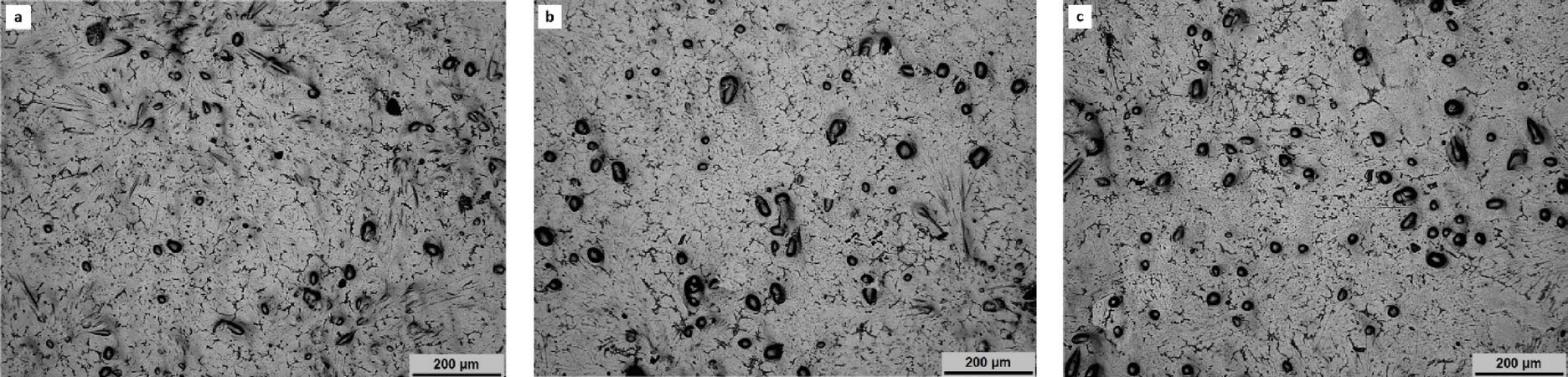
Optical micrographs of composites with (**a**) 2 wt%, (**b**) 4 wt% and (**c**) 6 wt% silica sand reinforcement particles.

The analysis of the composite microstructure provides valuable insights into the quality of casting and the distribution of reinforcement particles. During the fabrication of particulate-reinforced composites, two critical aspects must be considered: the uniform distribution of the particles during the pouring process and their alignment during solidification. Achieving superior mechanical properties relies on ensuring that the silica sand reinforcement is evenly dispersed throughout the AA6061 matrix. The micrographs captured using an upright metallurgical microscope reveal the consistent dispersal of silica sand particles within the AA6061 matrix, with no visible signs of porosities or voids at a current magnification of 4× (Fig. 7a-c).

**Fig. 8:**
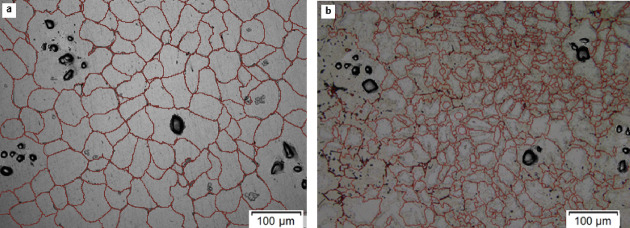
Microstructure of the AA6061-6% silica sand composite (**a**) before and (**b**) after thermomechanical treatment.

To assess the impact of LTMT on the microstructure of the composite, the grain size number (G) was evaluated using the Planimetric method in accordance with ASTM E112. Representative micrographs before and after processing are presented in Fig. 8. The AA6061–6% silica sand composite displayed a grain size number of 6.37 in the as-cast condition, which increased to 8.29 post-LTMT (15% deformation). This increase in the G value signifies notable grain refinement, as higher grain size numbers correspond to finer grains. Figure 8a shows a microstructure characterized by uniformly distributed coarse equiaxed grains. Following LTMT, the grains became more refined, as shown in Fig. 8b, resulting in reduced distortion and improved uniformity. Grain refinement was driven primarily by dynamic recrystallization, which breaks down larger grains into finer, more homogeneous grains. This structural evolution plays a key role in enhancing the mechanical properties, as finer grains serve as more effective barriers to dislocation motion, resulting in increased strength and hardness^30,31^.

To validate the presence of silica sand particles, Brinell hardness tests were conducted on the AA6061-silica sand composites. The results revealed increased hardness in the composites due to the incorporation of rigid dispersoids, which significantly contributed to the overall hardness^32,33^. Furthermore, increasing the silica sand content in the composites resulted in a gradual increase in hardness.

**Fig. 9:**
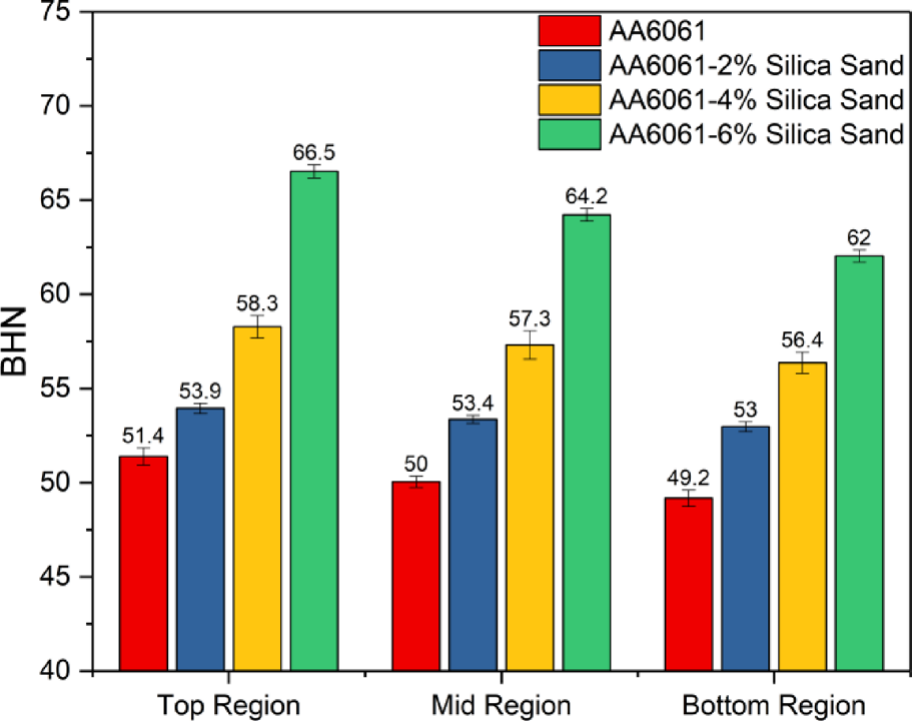
Brinell hardness values of the as-cast AA6061 and AA6061-silica sand composites.

The reinforcement particles play a key role in improving the hardness of the AA6061-silica sand composites. Hardness measurements were conducted for the AA6061 and AA6061-silica sand composites with various reinforcement levels (2%, 4%, and 6% by weight) across distinct zones: the bottom, middle, and top regions. Figure 9 presents the hardness data for both the base alloy and the composites, which were measured using a Brinell hardness testing machine at three different regions of the casting. The graph suggests that the consistent hardness observed across the composites strongly reflects the uniform distribution of Cu particles within the matrix. Furthermore, the calculated average standard deviation of the hardness values for each composite is 1.23, emphasizing the reliability and uniformity of the results.

Increasing the weight% of the reinforcement particles in the composites led to an increase in the dislocation density. This phenomenon occurs during solidification due to thermal mismatch between the reinforcement and the matrix, causing substantial internal stresses and strain that influence the microstructure and mechanical characteristics of the composites. The volume expansion of the reinforcement particles is accommodated by the plastic deformation of the matrix, resulting in a higher dislocation density. This elevated dislocation density enhances the resistance to plastic deformation, thereby increasing the hardness of the composites^34^. Additionally, during solidification, the presence of reinforcement particles in the aluminium alloy melt serves as heterogeneous nucleation sites, promoting finer grain structures and improving mechanical properties^35^.

### Assessment of the extracted reinforcement particles

**Fig. 10:**
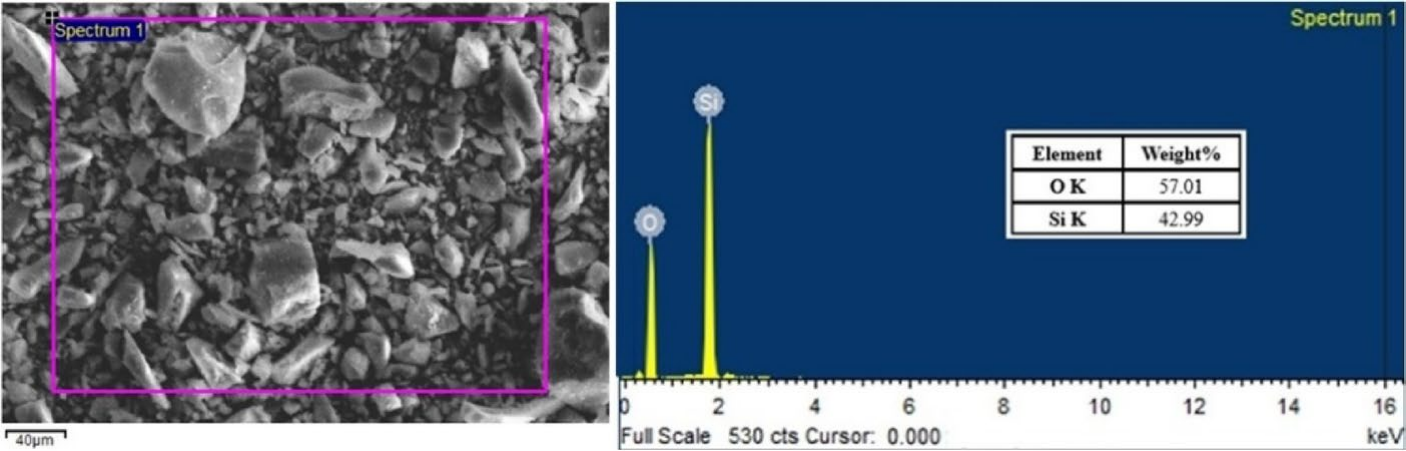
SEM and EDS of the reinforcement particles extracted from the AA6061-6% silica sand composites.

The extraction of particles from the composite was conducted to verify the presence of silica sand reinforcement at the expected weight percentages. Table 2 provides the actual and measured quantities of silica sand within the composite. The findings indicate a retention of at least 97% of the silica sand particles in all the composites tested. Figure 10 shows the SEM image and EDS of the extracted particles, clearly confirming that the particles obtained from the composite are indeed silica sand. Spectrum 1 confirms the incorporation of silica sand, as indicated by the high weight percentages of silicon (42.99%) and oxygen (57.01%), which are characteristic of silica sand (SiO₂). Thus, substantiating the intended material design, showcasing silica sand as integral phase in the aluminium matrix composite.

**Table 2:** Particulars of the wt% of silica sand particles in the composites.

Composite	Weight (g)			Silica sand in the composite (wt%)
	Composite specimen	Silica sand (Theoretical)	Silica sand (Measured)	
AA6061-2% silica sand	7.3150	0.1463	0.1438	1.97
AA6061-4% silica sand	6.6540	0.2662	0.2593	3.90
AA6061-6% silica sand	3.0711	0.1843	0.1803	5.87

### Assessment of density and void fraction

The density characteristics and void fractions of the AA6061 matrix composites reinforced with various weight percentages of silica sand (2%, 4%, and 6%) are presented in Table 3. The results indicate a slight decline in both the theoretical and actual densities with increasing silica sand content, which is attributed to the inherently lower density of the silica sand than that of the aluminum matrix. The void fraction increased from 1.846% for the 2% composite to 2.410% for the 6% composite. This increase in void content with increasing reinforcement level is likely due to inadequate wetting of the silica sand particles during processing.

**Table 3:** Void fractions of the AA6061-silica sand composites.

Composite	Theoretical density (ρ_t_) (g/cc)	Actual density (ρ_a_) (g/cc)	Void fraction (V_f_) (%)
AA6061-2% silica sand	2.711	2.661	1.846
AA6061-4% silica sand	2.710	2.655	2.017
AA6061-6% silica sand	2.709	2.644	2.410

### Peak hardness and behaviour of the ageing curves of the composites

**Fig. 11:**
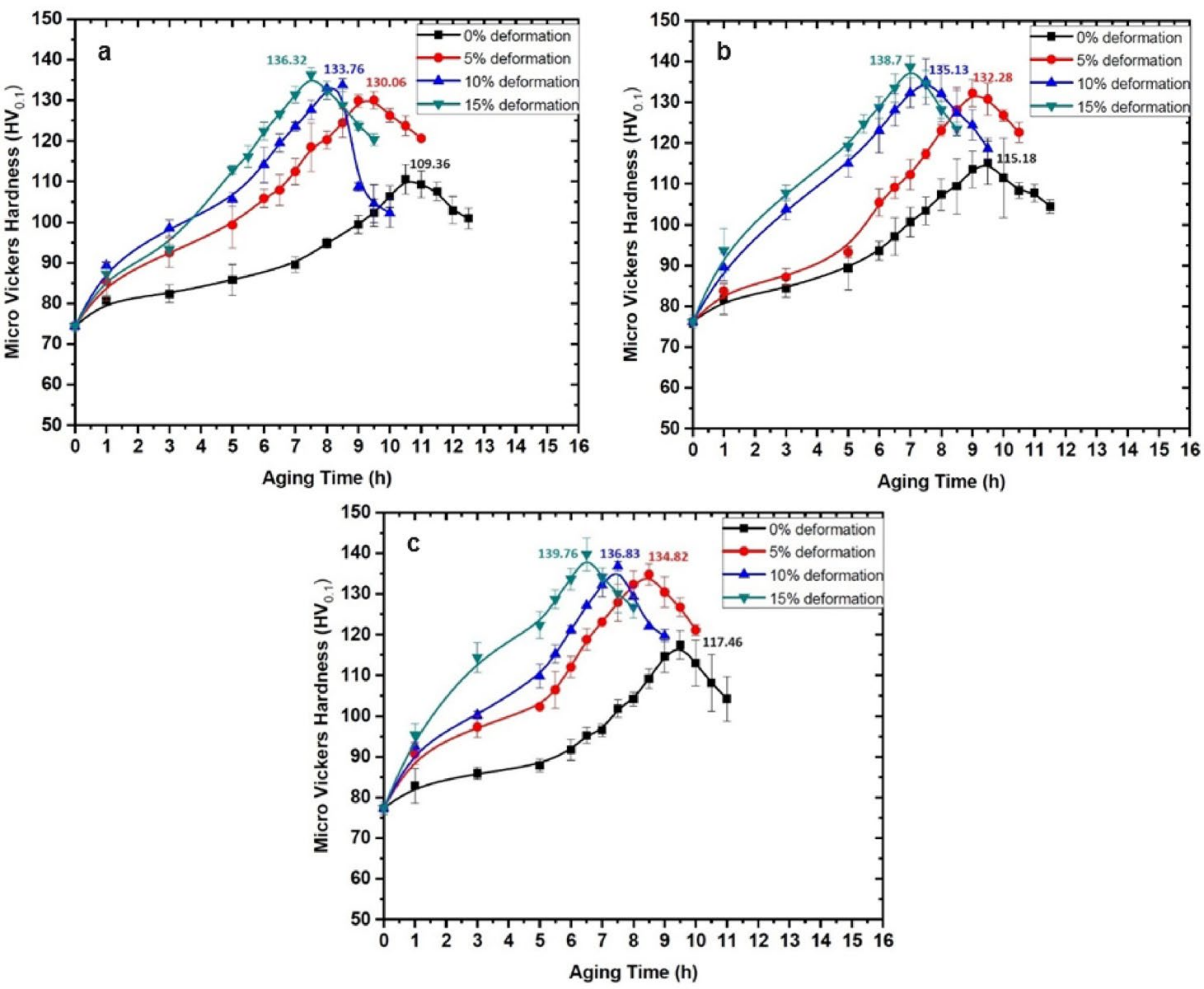
Vickers hardness vs. ageing time of (**a**) AA6061-2% silica sand, (**b**) AA6061-4% silica sand, and (**c**) AA6061-6% silica sand composites treated at 100 °C.

**Fig. 12:**
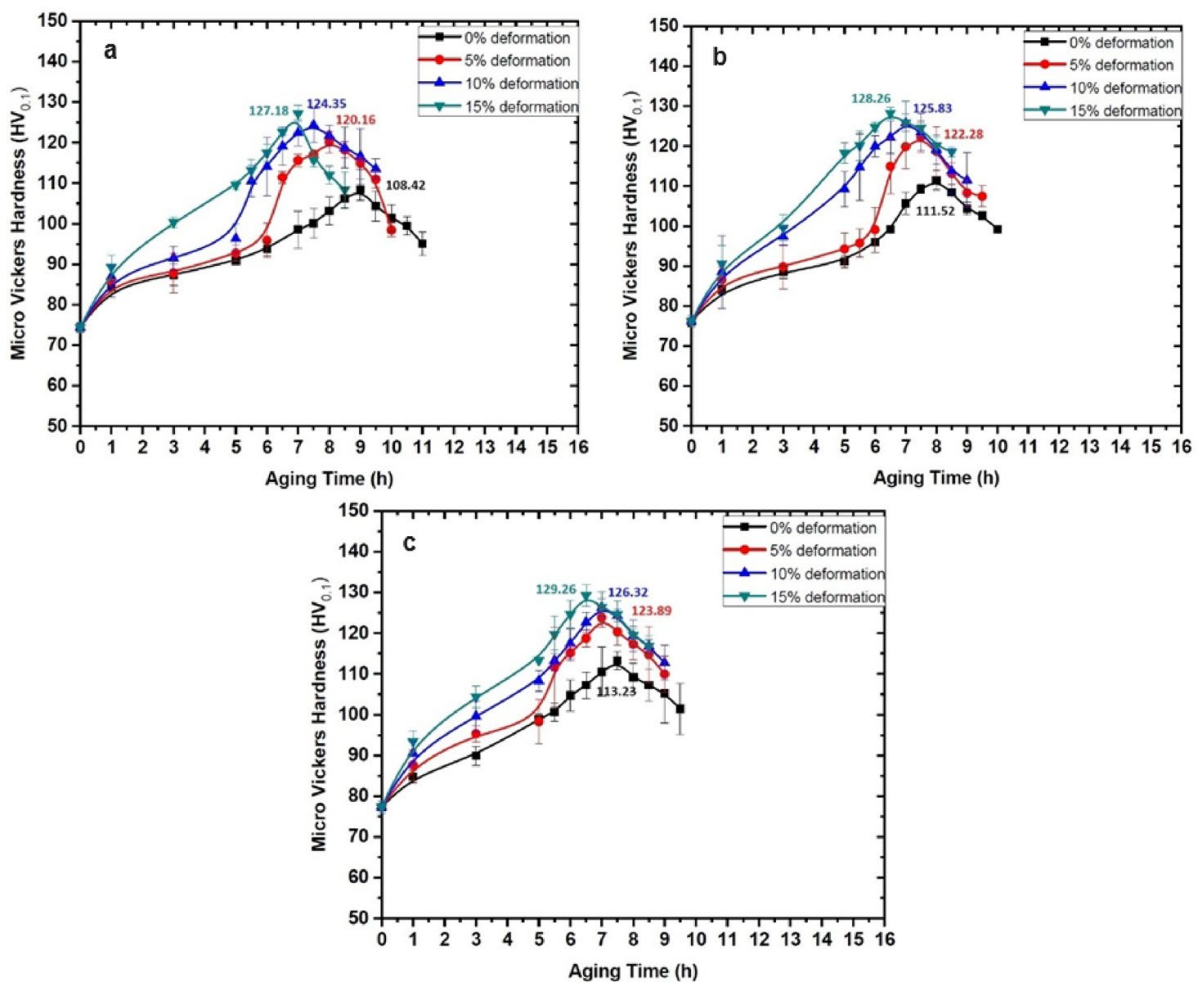
Vickers hardness vs. ageing time of (**a**) AA6061-2% silica sand, (**b**) AA6061-4% silica sand, and (**c**) AA6061-6% silica sand composites treated at 180 °C.

Vickers hardness tests were conducted to evaluate the hardness of AA6061-silica sand composites under three conditions: as-cast, age-hardened, and LTMT. The recorded hardness values varied within a range of ± 5 HV, ensuring consistency across the measurements. The hardness values of the as-cast composite specimens with 2, 4, and 6 wt% silica sand were 74.40, 76.20, and 77.34 HV, respectively, indicating that an increase in the silica sand content increased the hardness of the composites. Figures 11 and 12 display the hardness distributions over ageing time for the composites subjected to deformations of 0, 5, 10, and 15% at isothermal ageing temperatures (IATs) of 100 °C and 180 °C. Compared with the as-cast composites with 6 wt% silica sand, those aged at 100 °C and 180 °C presented peak hardness increases of 51% and 46%, respectively. Similarly, compared with age-hardened composites, the LTMT composites with 6 wt% silica sand, 15% deformation, and aged at 100 °C and 180 °C presented increases in peak hardness of 19% and 14%, respectively.

Increasing the reinforcement particle content elevates the dislocation density in composites due to thermal mismatch between the matrix and reinforcement during solidification. This mismatch generates internal stresses accommodated by plastic deformation of the matrix, leading to higher dislocation density, greater resistance to plastic deformation, and improved hardness^36,37^. Reinforcement particles also serve as heterogeneous nucleation sites, refining grains and enhancing mechanical properties^35,38^.

Property enhancement during age hardening depends on diffusion kinetics, governed by lattice strain and nucleation site density. Cold deformation increases dislocation density, stacking faults, and grain boundary areas, which act as nucleation sites for secondary phase precipitates^16,39^. During ageing, solute segregation produces metastable, solute-rich precipitates that maintain lattice coherency until a critical size is reached, resulting in peak hardness at the optimum isothermal ageing temperature^40,41^. Greater deformation provides more nucleation sites, producing finer precipitates with limited growth, thereby enhancing strength and hardness^42^. Increased dislocation density and lattice strain also accelerate ageing kinetics and contribute to higher resistance to plastic deformation^43,44^.

For the AA6061 composites, the combined influence of reinforcement particles and the precipitation of intermetallics during LTMT significantly increased the peak hardness. The highest peak hardness values, achieved with the shortest peak ageing times, were observed in the composites containing 6 wt% reinforcement treated thermomechanically under a maximum deformation of 15% at a lower IAT of 100 °C. The composite exhibited a peak hardness increase of 118% compared with that of the as-cast AA6061.

**Fig. 13:**
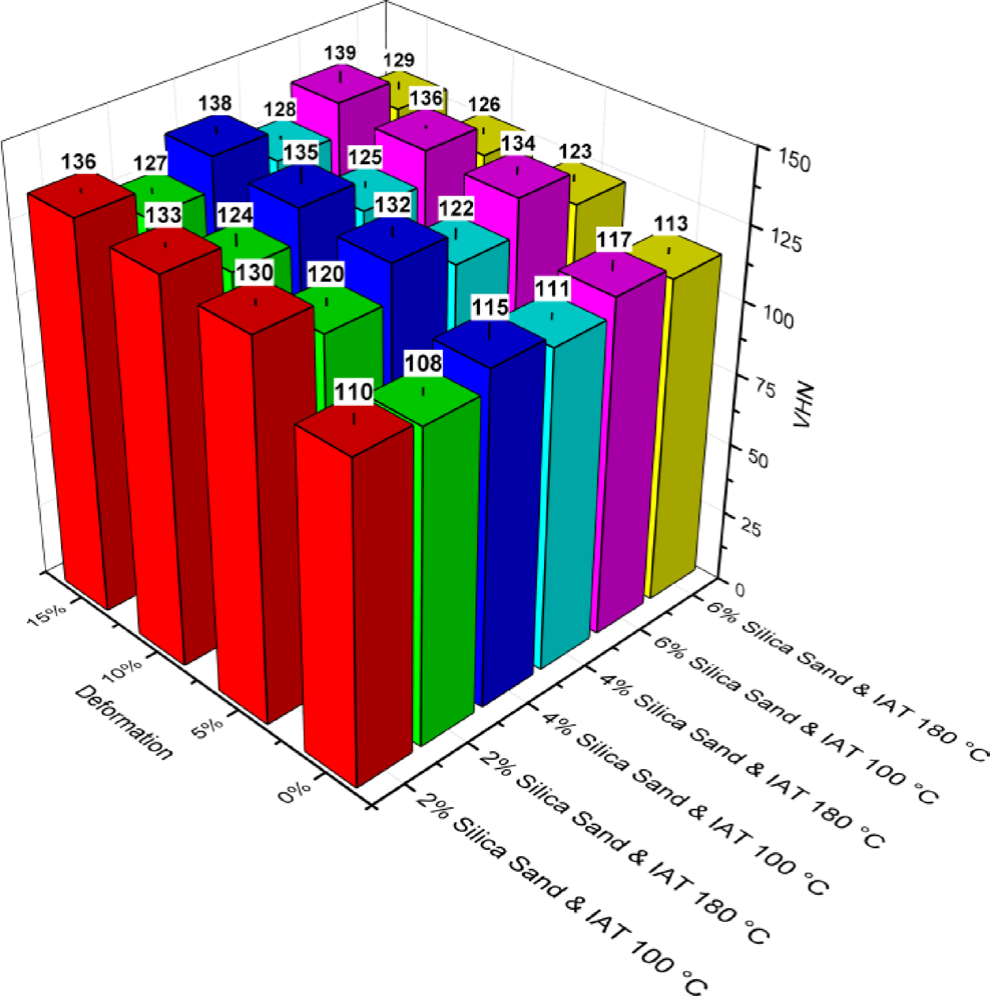
Vickers hardness values of the AA6061-silica sand composites under peak aged conditions.

Figure 13 highlights that even the addition of a small fraction (2 wt%) of reinforcement particles significantly enhances the hardness. This improvement is attributed to the inclusion of heterogeneous solid reinforcement particles in the melt, which promotes the formation of numerous nucleation sites, such as dislocations and stacking faults. However, the continued addition of reinforcement particles may not lead to a proportional increase in the number of nucleation sites, as observed in the initial case^45^.

The hardness distribution graphs demonstrated a steady increase in hardness over the ageing process, reaching a peak value before gradually declining. This behaviour is typical for aluminium alloys and aluminium-based composites during ageing at isothermal ageing temperatures (IATs) of 100 °C and 180 °C as reported by Kalaiselvan et al.^46^ and Rajasekaran et al.^47^.

### Tensile strength and fracture surface analysis

**Fig. 14:**
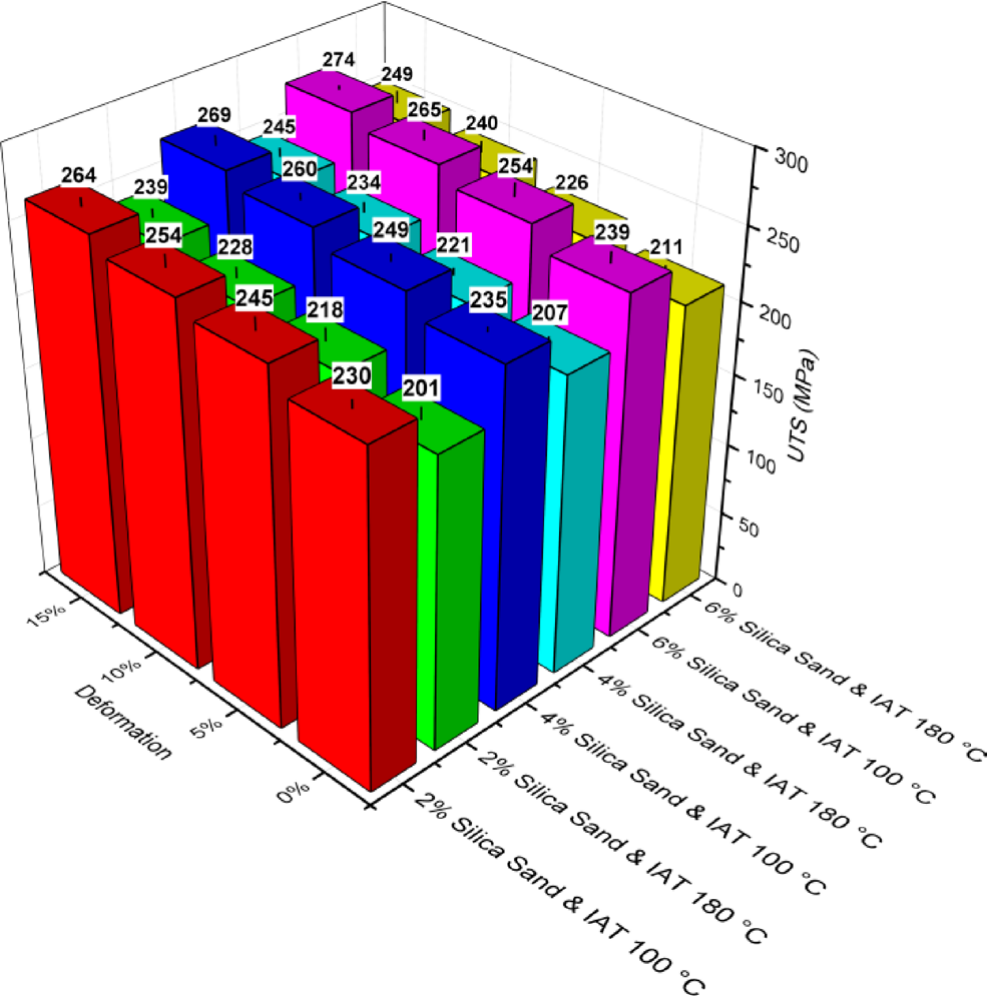
Ultimate tensile strength values of the AA6061-silica sand composites under peak aged conditions.

The tensile strength of the AA6061-silica sand composites was evaluated after age-hardening and thermomechanical treatment to assess the UTS along the rolling direction. The UTS exhibited a variation of ± 10 MPa. For the composite specimens in the as-cast conditions with 2%, 4%, and 6% silica sand contents, the UTS values were found to be 152, 160 and 169 MPa, respectively. Figure 14 clearly shows that there is a slight increase in the UTS with the inclusion of silica sand particles. In the case of MMCs, the presence of reinforcement particles serving as barriers to moving dislocations may cause a rise in UTS. The load-bearing ability of the reinforcements is the primary cause of the strengthening process of AA6061 composites. A strong resistance to the development of cracks in the composite results from the interplay between reinforcements and dislocations. At the matrix reinforcement interface, the differential thermal contraction caused by the residual thermal stresses generated around the reinforcement particles leads to strain. Many dislocations are generated in the matrix to accommodate the strain, substantially strengthening the matrix^48,49^.

Both age-hardened and LTMT-processed composites exhibit increased tensile strength. This improvement can be attributed to the strong interface between the silica sand reinforcement and the AA6061 matrix, which contributes to superior properties. Furthermore, the combination of cold rolling and ageing resulted in better tensile performance in the composites than in their as-cast counterparts. The observed increase in the strength of the composite may be attributed to the presence of silica sand particles, the formation of coherent Mg_2_Si during ageing that obstructs dislocation movement, and the greater degree of deformation induced by the rolling process^37,50^.

The UTS of composites is very sensitive to cold rolling and ageing. There was a minimum 42% increase in UTS for the age-hardened composites compared with the cast AA6061-silica sand composites with different amounts of reinforcements considering both IATs. At the lower IAT, the maximum UTS accomplished by the AA6061-6% silica sand composite with 15% deformation was 274 MPa (Fig. 14), an increase of 62 and 29% over those of the as-cast and age-hardened composites, respectively.

Fracture surface analysis primarily aims to determine the type of fracture exhibited by composite specimens. In aluminium alloys and aluminium matrix composites, ductile and brittle failure are the most common modes of fracture observed. Among the AA6061 composites, those reinforced with 6 wt% silica sand demonstrated superior hardness and tensile strength. Consequently, fracture analysis was conducted on composite specimens with 6 wt% reinforcement under as-cast and LTMT conditions.

**Fig. 15:**
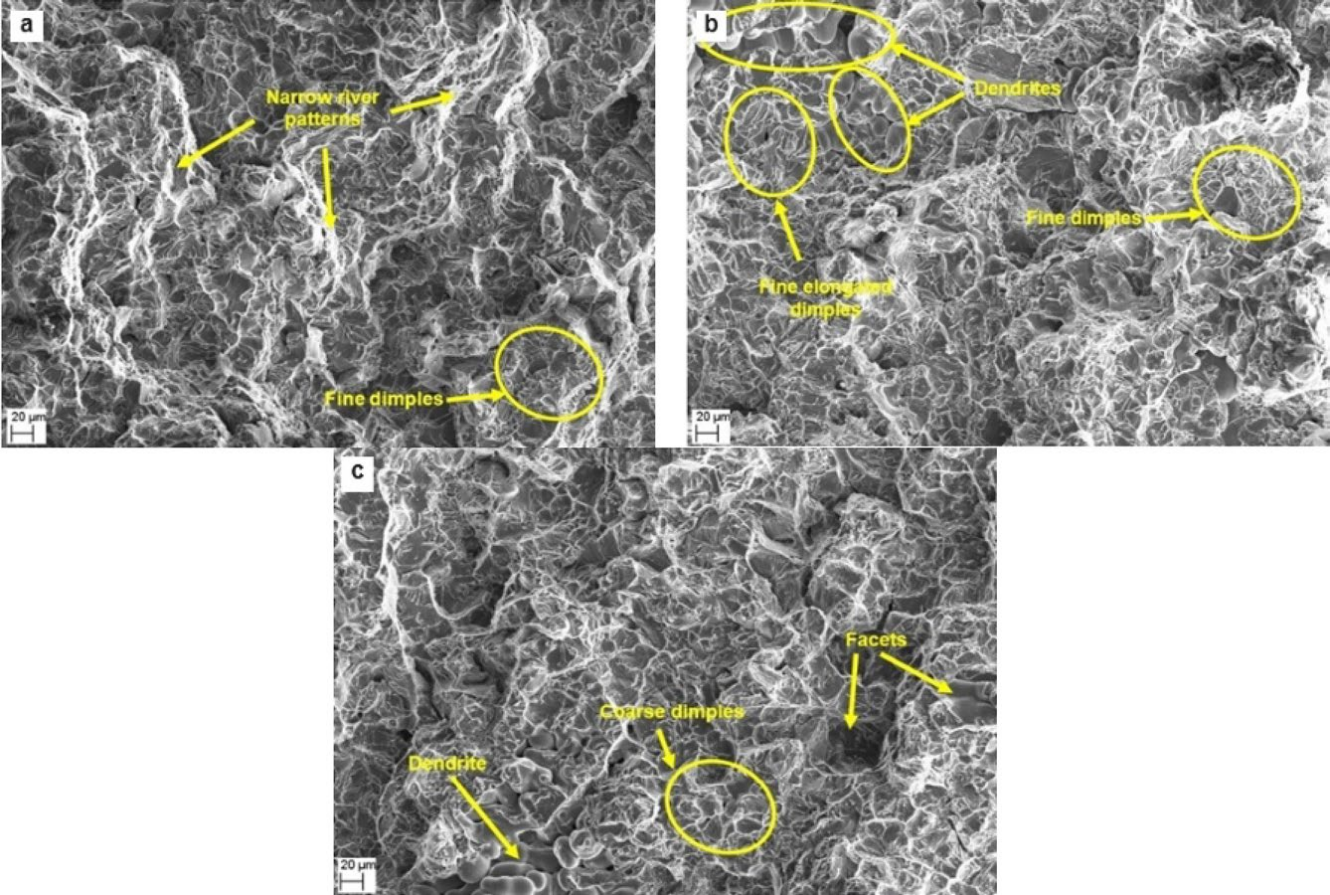
SEM fractographs of AA6061-6% silica sand composites in (**a**) as-cast conditions; thermomechanically treated with 15% deformation peak-aged at IATs of (**b**) 100 °C and (**c**) 180 °C.

The as-cast AA6061-6% silica sand composite fractographs (Fig. 15a) reveal that the failure of the composite occurred predominantly through the matrix alloy. Fine dimples exhibit ductile failure with strength, and narrow river patterns indicate plastic deformation of the matrix. The overall fracture mode was mixed in nature and dominated by ductile fracture. The AA6061-6% silica sand composite fractographs thermomechanically treated at 100 °C and 180 °C IATs (Fig. 15b-c) indicate increased strength and hardness. In localised regions, the fracture may be due to tear or shear, resulting in elongated dimples^51,52^, indicating increased tensile strength obtained through ageing. The well-defined dendrites indicate peak ageing, leading to increased hardness and strength. The facets accompanied by the spread of narrow river patterns exhibit brittle failure and local deformation during fracture.

## Conclusion

This experimental study aimed to incorporate naturally occurring ceramic particles in the fabrication of an AA6061 hybrid composite. Moreover, research has sought to introduce an innovative heat treatment process to increase the hardness and UTS of composites. The stir-casting method facilitated the production of AA6061-silica sand composites with evenly distributed reinforcement particles. The uniform distribution of the reinforcement particles in the composites was validated through microstructure analysis, extracted particle examination, and macro-hardness measurement using the Brinell hardness test. Analysis of the extracted particles confirmed 97% retention of the silica sand particles in the composites, as supported by the results from the SEM and EDS studies. Hardness measurements taken along the length of the fabricated composites with varying wt% reinforcement showed consistent values, further confirming the uniform distribution of the silica sand particles within the composites. Composites with 6 wt% silica sand, subjected to 15% deformation and aged at a lower IAT of 100 °C delivered exceptional results. Compared with the as-cast AA6061-6 wt% silica sand composites, the LTMT-processed composites with 15% deformation presented increases in peak hardness of 80% and 67% at 100 °C and 180 °C IATs, respectively. Compared with the age-hardened AA6061-6 wt% silica sand composites, the LTMT composites (15% deformation) presented increases in peak hardness of 19% and 14% at 100 °C and 180 °C IATs, respectively. Compared with those of the aged and as-cast composites, the UTS of the AA6061-6 wt% silica sand composite with the highest deformation increased by 4% and 62%, respectively, at a lower IAT. Maximum hardness and UTS values of 139.76 HV and 274 MPa were achieved in the AA6061-silica composites thermomechanically treated with 15% deformation at 100 °C IAT. Fracture analysis revealed a mixed fracture mode, predominantly brittle failure, across the as-cast, and LTMT-processed composites. The improved hardness and toughness of the LTMT composites were attributed to the dendrite-like structures observed in the fractographs.

## Data Availability

The datasets used and analyzed during the current study are available from the corresponding author upon reasonable request.
